# Effective mosquito and arbovirus surveillance using metabarcoding

**DOI:** 10.1111/1755-0998.12682

**Published:** 2017-05-15

**Authors:** J. Batovska, S. E. Lynch, N. O. I. Cogan, K. Brown, J. M. Darbro, E. A. Kho, M. J. Blacket

**Affiliations:** ^1^ Agriculture Victoria AgriBio Centre for AgriBioscience Bundoora Vic Australia; ^2^ School of Applied Systems Biology La Trobe University Bundoora Vic Australia; ^3^ Mosquito Control Laboratory QIMR Berghofer Medical Research Institute Brisbane Qld Australia

**Keywords:** bulk sample, Culicidae, *cytochrome c oxidase subunit I*, DNA barcoding, pooled samples, virus

## Abstract

Effective vector and arbovirus surveillance requires timely and accurate screening techniques that can be easily upscaled. Next‐generation sequencing (NGS) is a high‐throughput technology that has the potential to modernize vector surveillance. When combined with DNA barcoding, it is termed ‘metabarcoding.’ The aim of our study was to establish a metabarcoding protocol to characterize pools of mosquitoes and screen them for virus. Pools contained 100 morphologically identified individuals, including one Ross River virus (RRV) infected mosquito, with three species present at different proportions: 1, 5, 94%. Nucleic acid extracted from both crude homogenate and supernatant was used to amplify a 269‐bp section of the mitochondrial *cytochrome c oxidase subunit* I (COI) locus. Additionally, a 67‐bp region of the RRV E2 gene was amplified from synthesized cDNA to screen for RRV. Amplicon sequencing was performed using an Illumina MiSeq, and bioinformatic analysis was performed using a DNA barcode database of Victorian mosquitoes. Metabarcoding successfully detected all mosquito species and RRV in every positive sample tested. The limits of species detection were also examined by screening a pool of 1000 individuals, successfully identifying the species and RRV from a single mosquito. The primers used for amplification, number of PCR cycles and total number of individuals present all have effects on the quantification of species in mixed bulk samples. Based on the results, a number of recommendations for future metabarcoding studies are presented. Overall, metabarcoding shows great promise for providing a new alternative approach to screening large insect surveillance trap catches.

## 1 INTRODUCTION

Vector surveillance programmes are vital for the detection of invasive species and for the prevention and control of vector‐borne diseases. Effective surveillance programmes require rapid and accurate methods to identify trapped insect samples and also screen them for pathogens. Trapped insects are commonly identified using morphological traits, while pathogen screening may rely on culture from an insect pool, or molecular detection (Knope et al., 2013). These traditional methods can be time‐consuming, difficult to use for processing large quantities of insects, and require a variety of technical specialists (Besansky, Severson, & Ferdig, 2003).

DNA barcoding (Floyd, Abebe, Papert, & Blaxter, 2002; Hebert, Cywinska, Ball, & deWaard, 2003) is a molecular approach used to identify specimens to species and when combined with next‐generation sequencing (NGS) can determine the species present in mixed biological samples. This process is called metabarcoding and has a range of applications including biodiversity assessment (Yu et al., 2012), environmental monitoring (Carew, Pettigrove, Metzeling, & Hoffmann, 2013), characterizing microbiomes using 16S and 18S (Gibson et al., 2014), and vector surveillance (Kocher et al., 2017). Metabarcoding enhances vector surveillance by allowing the rapid identification of large numbers of insects, which could also be simultaneously screened for pathogens. Furthermore, due to the correlation between NGS reads and species abundance, metabarcoding has the potential to quantify the number of insects in a trap (Amend, Seifert, & Bruns, 2010; Carew et al., 2013; Zhou et al., 2013). The utility of metabarcoding in surveillance is dependent on its sensitivity and specificity, and also what DNA barcode databases are available.

Reference databases containing DNA barcodes corresponding to known species are essential for metabarcoding data analysis. The Barcode of Life Data Systems (BOLD) is the most comprehensive reference database currently available for insects and is regularly integrated with barcode data from the popular sequence depository GenBank (Benson, Karsch‐Mizrachi, Lipman, Ostell, & Sayers, 2009; Ratnasingham & Hebert, 2007). Almost 170,000 insect species have been barcoded to date (accessed July 2016); however, this is only a small fraction of the estimated 5,000,000 insect species in the world (Chapman, 2009). Sequences belonging to unknown taxa are a common problem in environmental DNA barcoding (Cowart et al., 2015; Pawlowski et al., 2011), highlighting the importance of a comprehensive reference database prior to commencing a metabarcoding study. The most commonly used marker for insect metabarcoding is *cytochrome c oxidase subunit I* (COI), although *cytochrome B* (CytB) and 16S rDNA have also been used (Carew et al., 2013; Kocher et al., 2017).

Mitochondrial genes such as COI and CytB are popular targets for barcoding due to their high copy number, conserved regions and lack of noncoding regions (Lin & Danforth, 2004; Yu et al., 2012). Certain nuclear genes can increase taxonomic resolution; however, they can be more difficult to amplify and are not well represented in reference databases. Using more than one marker is often beneficial in metabarcoding as it can alleviate biases in sequence recovery and improve the detection of low‐frequency species (Carew et al., 2013; Gibson et al., 2014). Diagnostic markers can also be added to screen for pathogens, thereby allowing the simultaneous detection of vector species and pathogens in mixed bulk samples.

Metabarcoding also benefits from the use of smaller barcoding regions. While the universal barcoding region for COI is typically 650 bp long, regions as small as 100 bp have been shown to successfully identify specimens to species (Meusnier et al., 2008). These ‘mini‐barcodes’ are more efficiently amplified in degraded samples and are easily sequenced by NGS technology (Hajibabaei, Shokralla, Zhou, Singer, & Baird, 2011). Due to the short read lengths of some NGS platforms, the size of the metabarcoding marker must be appropriate for the sequencing system being used. For instance, the Illumina HiSeq 2000 can only produce 100‐bp single‐end reads (Shokralla, Spall, Gibson, & Hajibabaei, 2012). Paired‐end reads can improve coverage of longer fragments and are joined during data analysis.

This study investigates the utility of metabarcoding in surveillance, using mosquitoes as an example. Mosquitoes are important vectors of disease and the targets of surveillance programmes worldwide (Eldridge, 2000). Vector surveillance programmes monitor the detection and abundance of indigenous species, as well as exotic and invasive species such as *Aedes aegypti* and *Aedes albopictus* (Kraemer et al., 2015), and are often coupled with arbovirus detection (Knope et al., 2013). Usually, the mosquito pools used for virus screening are small (<50 specimens) due to sensitivity issues associated with cell culture (Almeida et al., 2008; Ochieng et al., 2013). Furthermore, a subsampling system is often employed when a surveillance trap contains >1,000 mosquitoes due to the labour involved in identifying and screening large numbers of mosquitoes (Janousek & Olson, 2006). The development of a metabarcoding pipeline to upscale this process would significantly enhance vector surveillance programmes.

Schneider et al. (2016) describe a metabarcoding protocol to detect mosquito species in water samples; however, an approach on whole mosquitoes from traps that also includes virus detection has yet to be performed. This study utilizes metabarcoding to determine the species composition (meaning both the presence and abundance) of bulk mosquito samples, while also screening them for an arbovirus of public health significance: *Ross River virus* (RRV, Family *Togaviridae* Genus *Alphavirus*). The effect of laboratory protocol variables on the identification and quantification of mosquito species present in bulk samples and also on virus detection is specifically examined. These variables include sample centrifugation during DNA extraction, subsampling, primer design, PCR cycles and sample size.

## 2 MATERIALS AND METHODS

### 2.1 Mosquito collection

Three mosquito species were used in this study: *Aedes camptorhynchus*,* Anopheles annulipes* and *Aedes notoscriptus*. The first two species were collected from surveillance traps and identified as part of the Victorian Arbovirus Disease Control Program (VADCP) using previously described methods (Batovska, Blacket, Brown, & Lynch, 2016a). Both of these species were trapped in Lake Wellington, Victoria in January 2015. The *Ae. notoscriptus* specimens were obtained from the Queensland Institute of Medical Research (QIMR) Berghofer colony, which was established from wild‐caught material in Brisbane, Queensland in 2015. These mosquitoes were kept in an insectary at 25°C, ~80% relative humidity and 12/12‐hr photoperiod. Larvae were maintained with finely ground fish food (Tetramin Tropical Fish Food, Tetra), and adults were fed with 10% sucrose solution, ad libitum.

### 2.2 Infecting mosquitoes with Ross River virus

Ross River virus strain QML1 was isolated from human serum collected from Queensland, Australia (Jones, Lowry, Aaskov, Holmes, & Kitchen, 2010). Female *Ae. notoscriptus* mosquitoes (6–7 days old) were starved for 24 hrs and then transferred to 1‐L plastic feeding cups. Frozen stock of RRV (10^6.8^ TCID_50_/ml) was rapidly thawed and diluted 1:10 in defibrinated sheep's blood. Mosquitoes were fed with blood–virus mixture maintained at 37°C for 2 hrs by glass membrane feeders (Rutledge, Ward, & Gould, 1964). After feeding, mosquitoes were anaesthetized with CO_2_, and only fully engorged mosquitoes were maintained in new plastic feeding cups with 10% sucrose.

At 10 days postinfection, the mosquitoes were anaesthetized, and the bodies and legs of individuals were separated. Leg samples were tested for RRV using a cell culture enzyme‐linked immunosorbent assay (Oliveira et al., 1995) at QIMR Berghofer, Queensland. Of the 125 *Ae. notoscriptus* mosquitoes fed blood–virus mixture, 23 tested positive for RRV and these were sent to AgriBio, Victoria for use in the bulk pool samples.

### 2.3 Mosquito bulk pool preparation

A pool of mosquitoes was prepared consisting of 94 *Ae. camptorhynchus*, 5 *An. annulipes* and 1 *Ae. notoscriptus* mosquitoes. This is an artificial bulk sample that reflects species and proportions regularly found in mixed surveillance traps received by the VADCP. A second pool served as a viral negative, containing only 95 *Ae. camptorhynchus* and 5 *An. annulipes* mosquitoes. The exclusion of *Ae. notoscriptus* from this pool also functioned as a ‘species negative’ when sequenced with the other samples containing *Ae. notoscriptus*. Finally, to test the sensitivity of the method, a larger pool was prepared consisting of: 974 *Ae. camptorhynchus*, 25 *An. annulipes* and 1 *Ae. notoscriptus* mosquitoes. These pools are referred to as the 100 virus positive pool, 100 virus negative pool and 1000 pool, respectively (Table 1).

**Table 1:** Treatments and cycle numbers for each subsample within the three bulk mosquito pools. The COI read counts for species detected in each subsample are also shown

Sample			Read counts		
Pool	Treatment	Cycle number	*Aedes camptorhynchus*	*Anopheles annulipes*	*Aedes notoscriptus*
100 virus positive	Supernatant	27	166,658	16,568	561
			181,424	13,973	472
	Crude homogenate		226,774	18,604	566
			204,931	20,243	509
100 virus negative	Supernatant	30	289,891	7,130	0
		33	370,532	6,194	0
	Crude homogenate	30	260,248	8,045	0
			318,922	8,992	0
		33	391,736	11,604	0
			349,487	18,595	0
1000	Supernatant	30	274,864	3,407	0
		33	436,710	10,808	80
	Crude homogenate	30	294,744	2,909	11
		33	285,642	7,117	26

### 2.4 Nucleic acid extraction

The 100 virus positive and negative pools were homogenized in 1 ml of growth medium (Minimal Essential Medium (Life Technologies) supplemented with 7% v/v foetal bovine serum (Sigma Aldrich), 15 mm HEPES (Life Technologies), 100 μg/ml benzyl penicillin (CSL) and 5 μg/ml streptomycin B (Sigma Aldrich)) using 10 3 mm acid‐washed beads (Livingstone) and a 2010 Geno/Grinder (SPEX SamplePrep) at 1500 rpm. To investigate the effect on virus detection, four 200 μl subsamples were prepared: two were extracted from the crude homogenate, the other two were extracted from the supernatant after centrifugation for 1 min at 5,000 *g*. The remaining 200 μl homogenate could not be separated from the mosquito fragment and was not processed further.

Nucleic acid (including both DNA and RNA) was extracted from each of the 200 μl subsamples using 500 μl QuickExtract DNA Extraction Solution (Epicentre) according to the manufacturers’ instructions. The dsDNA concentration of the subsamples was quantified using a NanoDrop 1000 spectrophotometer (Thermo Scientific), and they were diluted to 40 ng/μl. All subsamples were stored at –20°C.

The 1000 pool sample was extracted as stated above, with the following alterations: 35 acid‐washed beads were used instead of 10; 10 ml of growth medium was added instead of 1 ml; and only a single 200 μl subsample was taken from the crude homogenate and the supernatant instead of duplicates (Table 1).

### 2.5 Primer design and amplicon production

All primers listed below were created with tails attached to them at the 5′ end: forward tail 5′‐ACACTCTTTCCCTACACGACGCTCTTCCGATCT; reverse tail 5′‐GACTGGAGTTCAGACGTGTGCTCTTCCGATCT. These tails serve as adapter primer sites during amplicon library preparation.

To amplify COI, the primer pair LCO1490 (Folmer, Black, Hoeh, Lutz, & Vrijenhoek, 1994) and the novel COI_R2 (5′‐TATTCGAGGAAAWGCYATATCWGG) were used, with tails as above. The reverse primer was designed using primer3 version 0.4.0 (Untergasser et al., 2012) utilizing a previously established COI barcode database of 27 mosquito species commonly found in Victoria, Australia (Batovska et al., 2016a). The primer was positioned in a conserved region of the COI gene, ensuring that the 269‐bp fragment created was located entirely within the barcode region used in the database. Degenerate bases were placed in the 3′ half of the primer to account for some of the known differences found between the 27 species. A neighbour‐joining tree of the 269‐bp region in COI was generated using sequences from the 27 species, and it was found that the differentiation between species was equivalent to what was found in Batovska et al. (2016a).

COI was amplified in a single specimen from each mosquito species to ensure the effectiveness of the tailed primer pair. Then, COI amplicons were produced from the mosquito pool samples. The 25‐μl PCR reactions contained: 14.7 μl 1 × bovine serum albumin (BSA); 5 μl of 5 × MyFi Reaction Buffer (Bioline); 1 μl of each 10 μm/L primer; 0.8 μl of MyFi DNA Polymerase (Bioline); and 2.5 μl of 40 ng/μl template DNA. The PCR programme was as follows: 94°C for 2 min; a maximum of 35 cycles of 94°C for 30 s, 49°C for 45 s and 72°C for 45 s; and 72°C for 1 min. Due to varying levels of amplification among the different pools, replicates of each subsample were used to compare a range of PCR cycle numbers (12–33) on a 2% w/v agarose gel. Only visible amplicons were used to ensure successful library preparation. The cycle numbers chosen for each pool were as follows: 100 virus positive pool (27 cycles); 100 virus negative pool and 1000 pool (30 and 33 cycles) (Table 1).

For RRV, the primer pair RRVE2F and RRVE2R (Hall, Prow, & Pyke, 2011) was used, also with tails attached to the 5′ end. Prior to amplicon production, each extraction was reverse transcribed using the SuperScript III First‐Strand Synthesis System (Invitrogen). The tailed RRVE2R primer was used for the cDNA synthesis reaction with 6.5 μl of undiluted extract. All manufacturer's instructions were followed; however, RNaseOUT was omitted from the protocol due to the high concentration of input RNA. The cDNA was used to amplify a 67‐bp region of the RRV E2 gene with the tailed RRV primers. The PCR reaction composition and programme is the same as above, with 40 cycles being used each time. The RRV amplicons were verified on a 2% w/v agarose gel.

### 2.6 Amplicon sequencing and data analysis

The COI and RRV amplicons were purified using AMPure XP beads (Beckman Coulter) at a 2 × beads ratio. PCR was used to attach unique 8‐bp barcodes and Illumina P5/P7 adapters onto the amplicons, and then, a further AMPure XP bead purification was performed with the same bead ratios. The samples were quantified by a 2200 Tape Station (Agilent Technologies) and pooled together in equimolar concentrations. The pooled sample was quantified using a Qubit 1.0 fluorometer (Thermo Scientific) and sequenced on a MiSeq platform (Illumina) with 2 × 250–300 bp reads. The 100 virus negative pool and 1000 pool were sequenced on a separate run to the 100 virus positive pool.

**Figure 1:**
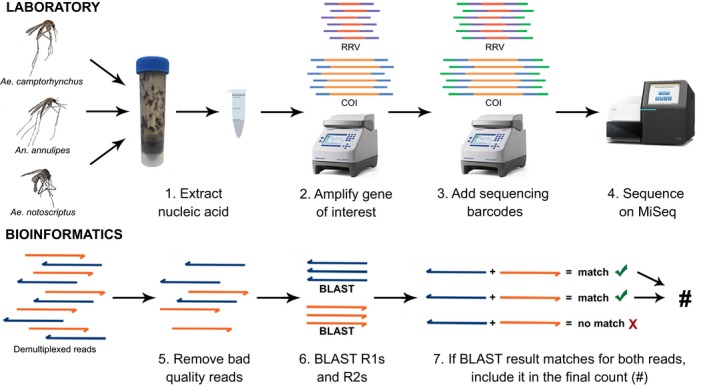
Overview of the metabarcoding method used to determine the species composition of mosquito pools and screen them for a virus. The bioinformatic pipeline shown is performed for each set of amplicon reads for each sample

Demultiplexed reads were quality trimmed using previously described parameters (Batovska, Cogan, Lynch, & Blacket, 2016b), and adaptors were removed using cutadapt version 1.9 (Martin, 2011). A blastn version 2.2.25+ search was performed on pairs of single‐end reads with the following parameters: alignment length >150 bp; percentage identical matches >95%; and a maximum of one aligned sequence per read to allow quantification. Custom databases were used for both COI and RRV data analyses. The COI database contained 111 COI sequences from 27 Victorian mosquito species acquired in Batovska et al. (2016a). All but one of the species (*Tripteroides tasmaniensis*) is represented by multiple COI sequences within the database. The RRV database consisted of the RRV strain QML1 genome (Jones et al., 2010). The BLASTN results for each pair of single‐end reads were compared and when both reads had the same species result the pair was counted, producing a final count for each species in each sample. A visual overview of both the laboratory protocol and bioinformatic pipeline is shown in Figure 1.

## 3 RESULTS

### 3.1 Species detection in mosquito pools

Both *Aedes camptorhynchus* and *Anopheles annulipes* were detected at varying proportions in all pooled samples (Tables 1 and 2). As expected, reads matching *Aedes notoscriptus* were detected in the 100 virus positive pool and 1000 pool, but not in the 100 virus negative pool. In the 1000 pool, *Ae. notoscriptus* was detected in only one of the two subsamples processed at 30 PCR cycles, accounting for 0.004% of the reads. However, at 33 cycles, *Ae. notoscriptus* was detected in both of the subsamples and accounted for 0.01–0.02% of the reads.

**Table 2:** Read proportions (%) for species detected in pooled samples. Proportions are shown as means with standard deviation, and the expected proportion in brackets

Pool	*Aedes camptorhynchus*	*Anopheles annulipes*	*Aedes notoscriptus*
100 virus positive	91.5 ± 0.9 (94)	8.2 ± 1.0 (5)	0.3 ± 0.04 (1)
100 virus negative	97.0 ± 1.1 (95)	3.0 ± 1.1 (5)	Absent (0)
1000	98.2 ± 0.8 (97.4)	1.8 ± 0.8 (2.5)	0.008 ± 0.008 (1)

### 3.2 Effect of sample centrifugation and PCR cycle on species composition

**Figure 2:**
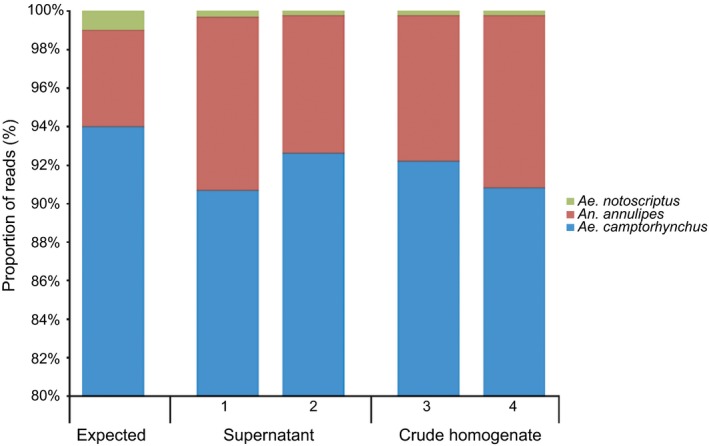
Comparison of the species composition for each subsample (1–4) from the 100 virus positive pool. Half of the subsamples (1–2) were extracted from supernatant, the other half (3–4) from crude homogenate. The expected species composition is also shown. Each bar represents 100 mosquitoes (proportion of *Ae. camptorhynchus* truncated; top 80% shown)

The crude homogenate and centrifuged supernatant subsamples from the 100 virus positive pool had no observable consistent difference in species composition (Figure 2). This was also true for the 100 virus negative pool subsamples and the 1000 pool subsamples.

**Figure 3:**
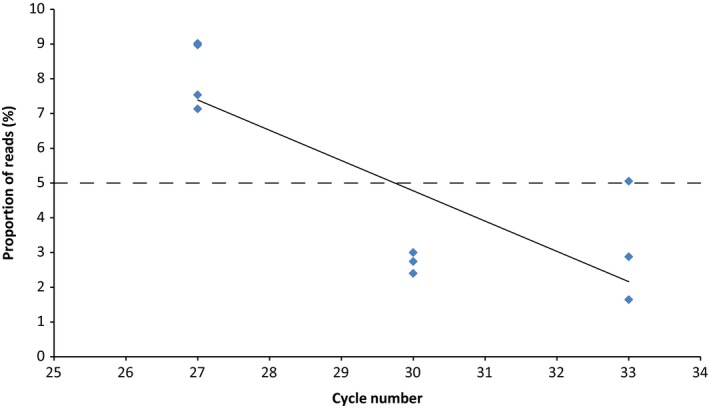
Comparison of PCR cycle number and the proportion of reads attributed to *Anopheles annulipes* from the 100 pool samples. The dashed line represents the expected proportion of reads. The solid line represents the linear regression line (*R* ^2^ = 0.63)

*Anopheles annulipes* was expected to account for 5% of the total reads for both the 100 virus positive pool and the 100 virus negative pool. At 27 cycles, there was a mean of 8.1% of *An. annulipes* reads and this percentage decreased as the PCR cycle number increased (Figure 3). The coefficient of determination for the relationship between cycle number and proportion of reads is 0.63.

### 3.3 Ross River virus detection

All mosquito pools containing a *Ae. notoscriptus* mosquito had a visible RRV amplicon when viewed on an agarose gel and produced RRV reads when sequenced. The RRV read number for positive samples ranged from 172,364 to 556,670 per subsample. The 100 virus negative pool also contained a small number of RRV reads, ranging from 0 to 121 reads per subsample. RRV sequence counts for each subsample can be found in Table S1, Supporting Information.

## 4 DISCUSSION

### 4.1 Species detection and quantification

This study corroborates the use of metabarcoding for species identification in bulk mosquito samples. Metabarcoding was able to detect all mosquito species (i.e., up to 3) present in each bulk sample tested, including the 1000 pool. Furthermore, species composition was consistent in subsamples (Figure 2), suggesting that one subsample from a bulk sample may be sufficient to characterize the species present. However, due to the sensitivity of NGS and the possibility of laboratory contamination, it is recommended multiple replicate subsamples are used to improve accuracy. At a minimum, samples with unexpected results should be re‐tested. It is also recommended that the bulk sample supernatant be subsampled rather than the crude homogenate, as they have no observable difference in species composition (Figure 2), and it prevents the transfer of large mosquito fragments.

The results indicate that even though sequence read number is correlated with mosquito abundance, it is not a completely accurate measure of species frequency (Table 2). This finding is consistent with other metabarcoding studies (Carew et al., 2013; Elbrecht & Leese, 2015; Piñol, Mir, Gomez‐Polo, & Agustí, 2015; Porazinska et al., 2009). Currently in surveillance programmes, the abundance of large trap catches is often estimated using weight, which can be affected by a number of variables including the size of the insects and the level of humidity in the catch (Kesavaraju & Dickson, 2012). As such, the approximate abundance derived from metabarcoding would still be useful for surveillance programmes.

While species abundance affects sequence read number, the number of PCR cycles used also has an effect on read numbers. Raising PCR cycle number increased the proportion of reads for the single *Aedes notoscriptus* specimen in the 1000 pool sample, thereby improving sensitivity, while in the 100 pool samples, it led to a decrease in the proportion of *Anopheles annulipes* reads (Figure 3). The inconsistent effect of PCR cycle number could be a result of primer bias. The reverse primer used in this study was specifically designed for mosquitoes, with the inclusion of degenerate bases to account for much of the interspecies variability. However, the universal LCO fragment was used as the forward primer (Folmer et al., 1994) and could have resulted in unequal species amplification due to mismatches. The use of universal primers for amplification of a range of species can cause variance in read number among taxa by several orders of magnitude (Elbrecht & Leese, 2015; Piñol et al., 2015). Approaches to reducing primer bias in metabarcoding studies include testing newly designed primers with in silico PCR to estimate taxonomic coverage (Clarke, Soubrier, Weyrich, & Cooper, 2014); using more conserved barcode regions (Kocher et al., 2017); designing multiple sets of primers that match specific taxonomic groups (Gibson et al., 2014); and utilizing PCR‐free shotgun sequencing pipelines (Zhou et al., 2013). Each of these methods has limitations, and the metabarcoding approach chosen needs to be based on the sensitivity and taxonomic resolution required for the target species.

### 4.2 Pathogen detection

In addition to species, metabarcoding also detected RRV in every pool containing an infected mosquito, indicating the potential of this method to improve not only species identification in surveillance programmes, but also virus detection. Ross River virus was detectable in both 100 and 1000 pools, highlighting the sensitivity of the method, and detection was not affected by centrifugation of samples prior to extraction. These results encourage testing for a wider range of arboviruses using group‐reactive primers (for example Eshoo et al., 2007; Kuno, 1998; Palacios et al., 2011). Due to the multiplexing capabilities of NGS, many large pools of vectors could be simultaneously screened for a panel of arboviruses, thereby improving the efficiency of virus screening. Metabarcoding could also be useful for investigating virus strain diversity within bulk pools due to the ability of NGS to capture all sequence variants in a sample (Beerenwinkel, Günthard, Roth, & Metzner, 2012). However, a larger fragment than the 67‐bp region used in this study would be required to provide meaningful strain diversity information.

Contamination appeared to be an issue in pathogen detection, with low numbers of RRV reads (up to 121 reads per subsample) detected in the 100 virus negative pool. The contamination occurred at a low frequency when compared to the positive subsamples (mean of 28.8 reads per 100 virus negative pool subsample compared to 376,125.7 reads per RRV‐positive subsample, Table S1, Supporting Information). Given that the 100 virus negative pool subsamples were sequenced on the same MiSeq run as the positive subsamples, it is likely that these contaminating reads are a result of imperfect demultiplexing, where individual sample indexes are misassigned. Imperfect demultiplexing has been reported in other studies and is attributed to mixed clusters on the flow cell and image analysis errors (Kircher, Sawyer, & Meyer, 2012; Nelson, Morrison, Benjamino, Grim, & Graf, 2014). Interestingly, no *Ae. notoscriptus* reads were detected in the 100 virus negative pool subsamples despite being run with other subsamples containing this species (Table 1). This could be due to the lower concentration of *Ae. notoscriptus* COI amplicons compared to RRV amplicons.

Due to the low level of contaminating reads in 100 virus negative pool subsamples when compared to the positive subsamples, a read count threshold could be applied during data analysis in order to solve the contamination problem. The read count thresholds would first need to be determined for each pathogen using positive samples prior to testing unknown samples, although our results indicate that this threshold is likely to be very low.

### 4.3 Future directions

Based on these results, there are a number of considerations that need to be addressed in future metabarcoding studies (Table 3). Factors such as primer design, nucleic acid extraction, PCR cycle number and analytical thresholds all need to be incorporated into the study design to achieve optimal surveillance results.

**Table 3:** Recommendations for metabarcoding projects based on the results from this study

Experimental factor	Recommendation
Primer design	Design taxon‐specific primers for the target organisms to maximize species recovery by reducing amplification bias.
DNA extraction	After homogenization, centrifuge the bulk sample and subsample from the supernatant. This allows for a more homogeneous sample and does not appear to affect species or pathogen detection
PCR cycle number	Optimize the PCR cycle number to the bulk sample size for all target species using known samples. Choose the cycle number that provides the required sensitivity and best quantification
Analytical thresholds	When sequencing multiple samples in one run, apply a read count threshold for positive results when analysing the data to account for possible imperfect demultiplexing

Further research is required to test pooled samples containing a wider range mosquito species, in order to better reflect the diversity of species seen in surveillance programmes. Additionally, it would be useful to expand the pathogen screening to include other arboviruses and arboviral families. The metabarcoding method could also be extended to other vector species, helping to improve regular surveillance programmes, and biosecurity response in the event of an exotic incursion (Comtet, Sandionigi, Viard, & Casiraghi, 2015).

As the number of mosquito reference barcodes increases, the databases used for the BLASTN search should include mosquito species from other regions, which could help to detect the spread of species to new geographic areas. Other data analysis methods could also be trialled with the mosquito metabarcoding data, such as clustering the reads into operational taxonomic units (OTUs), thereby allowing the detection of exotic species through the formation of novel clusters (Ji et al., 2013; Yu et al., 2012).

To improve the sensitivity and specificity of species characterization, the use of numerous barcoding markers could be explored. The use of additional markers relies on the establishment of more comprehensive and centralized databases, based on accessible, curated specimens (e.g., the ITS2 locus in Batovska et al., 2016b).

Sequence capture technology is an efficient approach to metabarcoding with multiple markers (Bragalini et al., 2014; Campana et al., 2016). This method involves using many sequence probes to capture species‐specific sequences, with one recent study designing 3,901 probes for a single NGS library preparation (Campana et al., 2016). The use of one reaction decreases the amount of required DNA, cost and time. Sequence capture could also potentially improve the bias found in metabarcoding where only one primer region is used, therefore requiring a lower degree of conservation among taxa. Capture technology could also be used to expand the breadth virus screening (Blouin et al., 2016).

When combined with other emerging technologies such as the Nanopore MinION, metabarcoding may be further utilized in biosecurity by offering “handheld barcoding” in the field (Bleidorn, 2016). This would allow real‐time trap characterization and pathogen detection without having to send samples back to a laboratory, which would be particularly useful during exotic incursions or viral outbreaks (Greninger et al., 2015).

## 5 CONCLUSIONS

The results of this study suggest metabarcoding is useful for the species characterization and pathogen detection of mosquito trap catches. We successfully detected a single RRV‐infected mosquito in a pool of 1000, emphasizing the sensitivity of the method and its utility in surveillance and biosecurity operations. While the accurate quantitative function of metabarcoding is currently limited due to the biases involved in primer use and PCR, we were still able to provide relative abundance information for the species contained in the pools. Due to the multiplexing capability of NGS technology, metabarcoding can lower the cost and time associated with sample processing in vector surveillance programmes (Ji et al., 2013). The efficiency of metabarcoding could be further improved with the addition of new technologies such as sequence capture and handheld sequencing. Metabarcoding shows great promise for modernizing vector surveillance programmes and improving the detection of pathogens of biosecurity significance.

## AUTHOR CONTRIBUTIONS

S.E.L., M.J.B. and N.O.I.C. conceived and designed the experiments. K.B. performed the mosquito morphological identification and bulk pool preparation. J.M.D. and E.A.K. provided the RRV‐infected *Ae. notoscriptus* mosquitoes. J.B. performed the DNA extractions, PCR experiments and sequencing. J.B. and N.O.I.C. analysed the sequencing data. J.B., M.J.B., S.E.L., N.O.I.C., E.A.K. and J.M.D. contributed to the drafting of the manuscript. All authors read and approved the final manuscript.

## DATA ACCESSIBILITY

Raw sequencing files for both COI and RRV amplicons are available at the National Centre for Biotechnology Information (NCBI) Short Read Archive (PRJNA343688). All the sequences used for BLASTN searches are available on GenBank (COI accessions: KU494977 to KU495080, KU495083 to KU495089; RRV accession: GQ433354).
