# Supplementary material for: Effective mosquito and arbovirus surveillance using metabarcoding
Source: Mol Ecol Resour. 2017 May 15;18(1):32–40. doi: 10.1111/1755-0998.12682 (PMC5811807; doi:10.1111/1755-0998.12682)
Supplement: Supplementary file 1 [file MEN-18-32-s001.docx]

**Supporting Information**

**Table S1:** RRV sequence counts for each subsample for each bulk mosquito pool. The different sample treatment for each subsample is also indicated.

| **Pool** | **Treatment** | **Read count** |
| --- | --- | --- |
| 100 virus positive | Supernatant | 351,199 |
|  |  | 172,364 |
|  | Crude homogenate | 426,005 |
|  |  | 556,670 |
| 100 virus negative | Supernatant | 7 |
|  |  | 1 |
|  | Crude homogenate | 121 |
|  |  | 36 |
|  |  | 0 |
|  |  | 8 |
| 1000 | Supernatant | N/A* |
|  |  | 213,393 |
|  | Crude homogenate | 513,799 |
|  |  | 399,450 |

* This sample was not sequenced as it did not pass library preparation quality control.
